# Identification of lncRNA competitively regulated subpathways in myocardial infarction

**DOI:** 10.3892/etm.2019.8210

**Published:** 2019-11-18

**Authors:** Xia Wu, Lili Sun, Ziliang Wang

Exp Ther Med 17: 3041-3046, 2019; DOI: 10.3892/etm.2019.7320

Subsequently to the publication of this article, the authors have realized that the first pair of authors listed on the paper, Xia Wu and Lili Sun, were not credited as joint first authors who made equal contributions to this study (asterisks should have been included alongside their names with the appropriate footnote to reflect this).

Therefore, the correct author affiliations for this article should have been presented as follows:

XIA WU^1*^, LILI SUN^1*^ and ZILIANG WANG^2^

^1^Department of Geriatrics, Daqing Oilfield General Hospital; ^2^Department of Cardiovascular Medicine, Daqing People's Hospital, Daqing, Heilongjiang 163316, P.R. China

*Contributed equally

The authors regret their oversight in this regard, and apologize for any inconvenience caused.

